# An Immunity-Based Anomaly Detection System with Sensor Agents

**DOI:** 10.3390/s91109175

**Published:** 2009-11-18

**Authors:** Takeshi Okamoto, Yoshiteru Ishida

**Affiliations:** 1 Department of Information Network and Communication, Kanagawa Institute of Technology/1030, Shimo-ogino, Atsugi, Kanagawa 243-0292, Japan; 2 Department of Knowledge-Based Information Engineering, Toyohashi University of Technology/1-1, Tempaku, Toyohashi, Aichi 441-8580, Japan; E-Mail: ishida@tutkie.tut.ac.jp

**Keywords:** immunity-based system, anomaly detection, intrusion detection, sensor agent, hidden Markov model, receiver operating characteristics

## Abstract

This paper proposes an immunity-based anomaly detection system with sensor agents based on the specificity and diversity of the immune system. Each agent is specialized to react to the behavior of a specific user. Multiple diverse agents decide whether the behavior is normal or abnormal. Conventional systems have used only a single sensor to detect anomalies, while the immunity-based system makes use of multiple sensors, which leads to improvements in detection accuracy. In addition, we propose an evaluation framework for the anomaly detection system, which is capable of evaluating the differences in detection accuracy between internal and external anomalies. This paper focuses on anomaly detection in user's command sequences on UNIX-like systems. In experiments, the immunity-based system outperformed some of the best conventional systems.

## 1. Introduction

Intrusion detection on the Internet is a pressing problem for the Information Society. Intrusion detection in user behavior can be divided into two approaches: misuse detection and anomaly detection. Misuse detection systems use sensors to monitor and scan for known misuses, while anomaly detection systems have sensors that monitor and detect deviations from normal behavior. The misuse detection system sensor can detect misuse by a malicious user. However, the sensor would have difficulty in detecting unknown types of misuse because of a lack of information regarding the specific misuse. In an anomaly detection system, the sensor has the potential to detect misuse by evaluating deviations from normal behavior, even if the specific details of the type of misuse are unknown. However, anomaly detection systems sometimes gives rise to false alarms, which can result in “the boy who cried wolf” syndrome. The objective of anomaly detection is to reduce missed alarms without giving rise to false alarms. This paper focuses on anomaly detection of a masquerader using someone else's account on a multiuser system, such as a UNIX-like system.

Studies on masquerader detection have employed various approaches, including incremental probabilistic action modeling (IPAM) [1], hidden Markov models (HMM) [2,3], the uniqueness approach [4], *etc.* The uniqueness method outperforms the hybrid multistep Markov method [5], Bayes 1-step Markov method [6], compression method [7], sequence-match method [8], and IPAM method [1] with a false alarm rate between 1% and 5% [7]. These methods have been restricted to systems using a single sensor. One drawback of the single sensor is that many false alarms arise when a valid user carries out new operations they have never performed previously.

In this paper, we propose an immunity-based anomaly detection system with multiple sensor agents based on the specificity and diversity of the biological immune system, in which each immune cell has a unique receptor that has a high affinity for only specific antigens. Similarly, each of our agents has a unique sensor, which reacts strongly to the behavior of a specific user. When a user types a command sequence, all the agents check their own score of the command sequence by their sensor. On the basis of all the scores, one of the agents determines whether the user is a masquerader or not. That is, our approach makes use of multiple sensors rather than a single sensor, which leads to an improvement in masquerader detection accuracy.

In performance evaluation, detection accuracy has been evaluated with no distinction between users internal and external to a LAN. In general, anomaly detection methods use the information of only internal users. This causes a difference in detection accuracy between internal and external users, because the detection accuracy is proportional to the amount of user behavior information available. However, the difference has been ignored in performance evaluations of anomaly detection. Here, we introduce an evaluation framework that is capable of evaluating the differences in detection accuracy between internal and external users.

Artificial immune systems for computer security can be divided roughly into three types [9]: hybrid approaches combined with multiple conventional detection methods [10-12], approaches inspired by the mechanism of negative selection in the thymus [13-17], and approaches motivated by the danger theory [18-21]. Our system is related to those using the second approach. These systems randomly generate short sequences, and those matching normal sequences, *i.e.*, self, such as system calls, are eliminated in advance. The remaining sequences are defined as anomalous sequences, *i.e.*, nonself, and these sequences are used for anomaly detection. However, such systems reported previously do not outperform the single-sensor method using the HMM [16]. The important difference in intrusion detection between our system and those reported previously is the reference information used for detection. Previous systems referred only to nonself information, while our system refers to both self and nonself information. This reference to self information contributes to a reduction in false alarms.

The remainder of this paper is organized as follows. Section 2 presents a review of the framework of the conventional anomaly detection method, and Section 3 presents the new immunity-based anomaly detection system with multiple sensor agents. Section 4 introduces a new evaluation framework for the immunity-based anomaly detection system. Section 5 presents the results of our experiments to determine the effectiveness of the immunity-based method, and discusses how to further improve detection accuracy. Finally, we conclude this paper in Section 6.

## 2. Anomaly Detection

The aim of anomaly detection is to prevent intruders and masqueraders from abusing computers and networks. Anomaly detection detects deviation from normal behavior. Therefore, in contrast to misuse detection, anomaly detection has the potential to detect unknown anomalous behavior.

An anomaly detection system requires construction of a profile for each user in advance. The profile accounts for the normal behavior of a user. The user behavior is expressed by an operation sequence, such as a command history, web history, *etc.* To date, such profiles have been constructed using various algorithms: HMM, IPAM, Bayes 1-step Markov method, *etc.* Constructing the profile requires operation sequences, called “training data” in the field of artificial intelligence, which are not evaluated as “test data”. The profile yields the likelihood that an operation sequence is performed by the original user, corresponding to the original user's profile.

After construction of user profiles, the system moves on to the test stage, in which it monitors operation sequences and computes the likelihood of the operation sequence with the profile corresponding to the account on which the command sequence is performed. If the likelihood is above a certain threshold, the operation sequence is considered normal, *i.e.*, the user who performed the operation sequence is the legitimate owner of the account on which the operation sequence was performed. If not, the operation sequence is deemed abnormal, *i.e.*, the user is not the legitimate owner of the account.

Here, we review how to discriminate between normal and abnormal operation sequences. In the user discrimination stage, the anomaly detection system computes the likelihood of an operation sequence with a single profile corresponding to an account on which a user performed the operation sequence. The other profiles are not used at all.

The profile of the account on which the user performed the operation sequence provides the likelihood that the user is a legitimate user. All the other profiles provide the likelihood that the user is not a legitimate user. That is, all the other profiles have the potential for detecting non-legitimate users, such as intruders or masqueraders. Hence, we propose a new framework for anomaly detection using not only the profile of the account on which the user performed the operation sequence, but also all the other profiles.

## 3. Immunity-Based System for Detecting Masqueraders

### 3.1. Definitions of “Self” and “Nonself”

The heart of the biological immune system is the ability to distinguish between “self” (*i.e.*, the body's own molecules, cells, and tissues) and “nonself” (*i.e.*, foreign substances, such as viruses or bacteria). Similarly, command sequences executed by a user on his/her own account are defined as “self”, and all other sequences are defined as “nonself”. For example, if one user executes commands on his/her own account, the command sequence is “self”. If another user executes commands on someone else's account, the command sequence is “nonself”. Such a user is defined as a masquerader or an intruder, regardless of whether the user's actions are malicious.

In the immunity-based anomaly detection system, command sequences per user in the training data belong absolutely to “self”. The command sequences are used for constructing a profile per user (using the algorithm described in Section 3.2). The profile yields the probability that the command sequence belongs to “self”. Based on this probability, the system classifies the command sequence as either belonging or not belonging to “self” as described in Section 3.3.

### 3.2. Generation of Agents

Immune cells have a unique receptor with high affinity for only specific antigens. Similarly, our immunity-based system generates a user-specific agent for every user, *i.e.*, every account. Each agent has a unique sensor to represent the probability that the command sequence is performed by the original user. The probability is expressed by a score, which is derived from the detection method, *i.e.*, HMM, IPAM, the Bayes 1-step Markov method, *etc.* We chose the HMM method because previous studies indicated that the HMM performs well [3,16]. We formally define the following notation for the HMM [22]:
*L*Length of a command sequence*K*Number of command sequences*O* = {*O*^1^, *O*^2^,…, *O^K^*}Command sequences$O^{k}=\left\{o_{1}^{k},o_{2}^{k},\ldots ,o_{L}^{k}\right\}$Command sequence where 
$o_{l}^{k}$ is the l^th^ command in the k^th^ command sequence*N*Number of hidden states for the HMM*M*Number of commands used in training data*V* = {*ν*_1_, *ν*_2_,…, *ν_m_*,…, *ν_M_*, *ν_M_*_+1_}Discrete set of commands used in training data, such as “ls”, “gcc”, “less”, *etc.* (only *ν_M_*_+1_ represents all commands not seen in the training data), where *m* is an identification number and 1 ≤ *m* ≤ *M*+1*A* = {*a_ij_*}State transition probability where *i, j* is a state and 1≤ *i, j* ≤ *N**B* = {*b_j_* (*m*)}Command probability distribution in state *j* where *m* is a command number and 1 ≤ *m* ≤ *M*+1 and 1 ≤ *j* ≤ *N**π* = {*π_i_*}Initial state distribution where *i* is a state and 1 ≤ *i* ≤ *N**P* (*O^k^*|*λ*)Likelihood of the command sequence *O^k^* with the profile *λ*

The parameter of the HMM is given by *λ* = [*A, B, π, V*], and corresponds to a single “user profile”, which accounts for user behavior. The parameter *V* is determined by command sequences seen previously in the training data as described in Section 5.1. Note that *v*_M+1_ represents all commands not previously seen in the training data.

The parameter *A, B, π*, is estimated from the training data composed of command sequences obtained previously from each user. The parameter estimation is conducted using the following Baum-Welch algorithm [22].

1. Generate the parameter *A, B, π* randomly.
2. Estimate the parameter *Ā, B̄, π̄* using the following equations:
  (1) $${\bar{a}}_{\text{ij}}=\frac{\sum_{k=1}^{K}\frac{1}{P(O^{k}|\lambda )}\sum_{l=1}^{L-1}\alpha_{l}^{k}(i)a_{\text{ij}}b_{j}\left(o_{l+1}^{k}\right)\beta_{l+1}^{k}(j)}{\sum_{k=1}^{K}\frac{1}{P(O^{k}|\lambda )}\sum_{l=1}^{L-1}\alpha_{l}^{k}(i)\beta_{l}^{k}(i)}$$
  (2) $${\bar{b}}_{j}(m)=\frac{\sum_{k=1}^{K}\frac{1}{P(O^{k}|\lambda )}\sum_{l=1,s.t.o_{l}^{k}=\nu_{m}}^{L-1}\alpha_{l}^{k}(j)\beta_{l}^{k}(j)}{\sum_{k=1}^{K}\frac{1}{P(O^{k}|\lambda )}\sum_{l=1}^{L-1}\alpha_{l}^{k}(j)\beta_{l}^{k}(j)}$$
  (3) $${\bar{\pi }}_{i}=\frac{\sum_{k=1}^{K}\frac{1}{P(O^{k}|\lambda )}\sum_{j=1}^{N}\alpha_{1}^{k}(i)a_{\text{ij}}b_{j}\left(o_{2}^{k}\right)\beta_{2}^{k}(j)}{\sum_{k=1}^{K}\frac{1}{P(O^{k}|\lambda )}\sum_{i=1}^{N}\alpha_{1}^{k}(i)\beta_{1}^{k}(i)}$$
  where:
  (4) $$\alpha_{1}^{k}(i)=\pi_{i}b_{i}(o_{1}^{k})$$
  (5) $$\alpha_{l+1}^{k}(j)=\sum_{i=1}^{N}\alpha_{l}^{k}(i)a_{\text{ij}}b_{j}(o_{l+1}^{k})$$
  (6) $$\beta_{L}^{k}(i)=1$$
  (7) $$\beta_{l}^{k}(i)=\sum_{j=1}^{N}a_{\text{ij}}b_{j}\left(o_{l+1}^{k}\right)\beta_{l+1}^{k}(j)$$
3. Iterate Step 2 using *Ā, B̄, π̄* in place of *A, B, π* until the iteration count reaches 1,000.

In this algorithm, *b_j_*(M + 1) is estimated to be 0.0, because there are no commands corresponding to *v*_M + 1_; *i.e.*, the likelihood *P(0^k^|λ)*, which will be described in the next paragraph, would produce zero probability for any sequence that includes *v*_M + 1_ even though *v*_M + 1_ is not always used by masqueraders. Such zero probability significantly degrades detection accuracy. To solve this problem, we replace *b̄_j_*(*m*) with the minimum probability, δ, if *b̄_j_*(*m*) is less than δ in Step 2 based on the method of parameter thresholding [22].

The agent can compute the likelihood *P(0^k^|λ)* of the command sequence *0^k^* with the profile λ. The likelihood *P(0^k^|λ*) represents the probability that the command sequence *0^k^* was performed by the original user corresponding to the agent *i.e.*, the profile λ. The likelihood *P(0^k^|λ*) is obtained by:

(8) $$P(O^{k}|\lambda )=\sum_{i=1}^{N}a_{L}^{k}(i)$$

The agent would compute a high likelihood, *i.e.*, a high score, for only the sequences of the original user corresponding to the agent. That is, the agent would recognize a specific user.

### 3.3. Discrimination of Self and Nonself

Our immunity-based system has a user-specific sensor agent for every account. All the agents on a computer communicate with each other, and one of the agents makes a decision on whether the command sequence is “self”, *i.e.*, the command sequence is performed by the original user.

The discrimination process is as follows:

Step 1. Each agent monitors commands on its own account until the length of the command sequence reaches *L*.
Step 2. The agent of the account on which the length of the command sequence reaches *L* is activated.
Step 3. The activated agent shares the command sequence with all the other agents.
Step 4. All agents compute their own score of the command sequence.
Step 5. The activated agent computes the effective threshold, *Min* + (*Max - Min*) × *Th*, where *Min* is the minimum score of all scores, *Max* is the maximum score of all scores, and *Th* is the percentage difference between *Min* and *Max*.
Step 6. The activated agent compares its own score, *X*, with the effective threshold, *Y*.
Step 7. If *X* ≥ *Y*, the activated agent classifies the command sequence as normal, *i.e.*, self. Otherwise, the agent classifies the command sequence as abnormal, *i.e.*, nonself. Exceptionally, provided that *X* is equal to the minimum value of *P*(*O^k^*|*λ*), *i.e., σ^L^*, the sequence is regarded as abnormal. Conversely, the sequence is regarded as normal if *X* is equal to the maximum value of *P*(*O^k^*|*λ*), *i.e.*, 1.0.
Step 8. If the activated agent decides that the command sequence is abnormal, it raises an alarm to a systems administrator.
Step 9. The activated agent returns to a normal state, and the process returns to Step 1.

**Figure 1.**
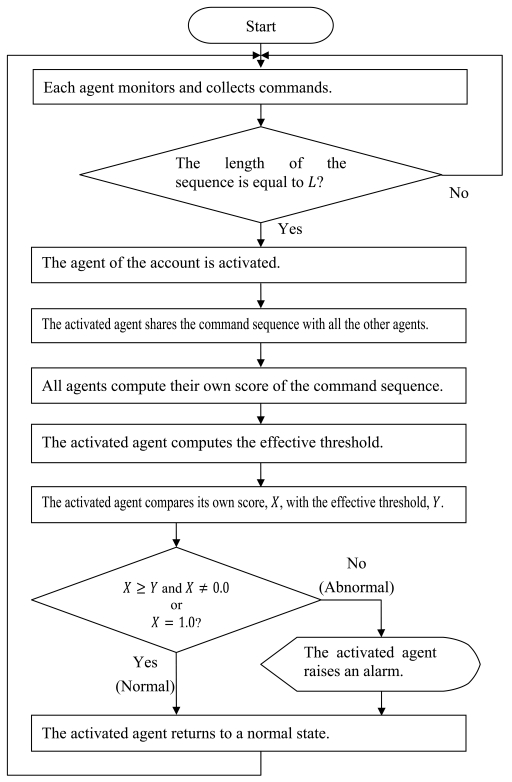
Flow diagram for discrimination of self and nonself.

**Figure 2.**
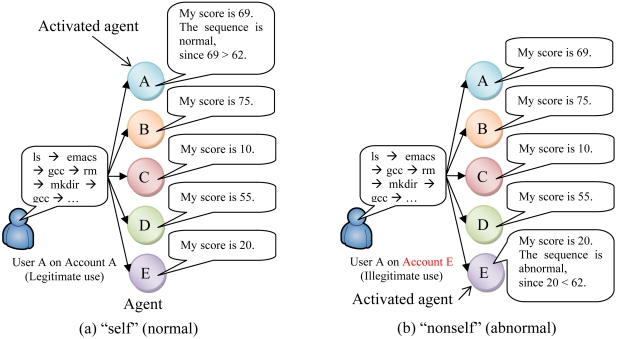
Discrimination of self (a) and nonself (b). There are five users on a computer. An agent is generated on every account. Let the threshold *Th* be 80%. When these agents compute scores of 69, 75, 20, 55, and 10, the effective threshold value is 62 (= 10 + (75 - 10) × 0.80. (a) When user A executes a command sequence on his/her own account, agent A of account A is activated, and the activated agent decides that the command sequence is normal as the score of the activated agent is ≥ 62. (b) When user A executes a command sequence on account E, agent E of account E is activated, and the activated agent decides that the command sequence is abnormal as the score of the activated agent is < 62.

The flow diagram is also shown in Figure 1. Examples of user discrimination are shown in Figure 2.

## 4. Evaluation Framework for Detection Accuracy of the Immunity-Based System

In the human body, nonself is divided into external nonself, such as bacteria and viruses, and internal nonself, such as tumor cells. Similarly, masqueraders are divided into masqueraders internal and external to the organization. For example, an internal masquerader may be a coworker who uses a computer without permission in some way, e.g., while the authentic user has left their desk. An external masquerader is a complete stranger to the organization who logs into a remote account on a remote server, such as the SSH or telnet server, by remotely cracking an account's password or exploiting some vulnerability of the remote server. Hence, it is easy to monitor and collect the behavior of internal masqueraders, but it is more difficult to monitor external masqueraders because such users rarely intrude and often destroy all evidence of their intrusion. The difference in amount of information for user behavior between internal and external masqueraders may affect the accuracy of detection between the two types of non-legitimate user. Particularly, our system could be advantageous for the detection of internal masqueraders, because it makes full use of information regarding such non-legitimate users. Therefore, user behavior data for evaluation should be divided into internal and external user behavior data.

Due to the difficulty in collecting external user data, the user data collected within the organization may be logically divided into internal and external user data. In this case, all combinations of internal and external users should be evaluated, as some users can be detected easily, while others will be more difficult. The total number of combinations may be extremely high in organizations with many users. Therefore, some of the combinations may be omitted to reduce the computation time.

Evaluation of detection accuracy requires user behavior data for both normal users and masqueraders. The normal user data can be collected easily from the organization, but collecting masquerader data is more difficult. Thus, the masquerader behavior is simulated by testing one user's behavior against another user's profile. This simulation is based on the assumption that other user's behavior will seem unusual for the original user.

The metrics of detection accuracy are based on the false alarm rate (*i.e.*, false positive rate) and missed alarm rate (*i.e.*, false negative rate); the former is the rate at which the system falsely regards a legitimate user as a masquerader, while the latter is the rate at which the system falsely regards a masquerader as a legitimate user.

We formulated the average false alarm rate as follows. Let *FA_ij_* be the number of false alarms for the combination *i* and the user *j*. Then, the false alarm rate for the combination *i* and the user *j* is $\frac{{\text{FA}}_{\text{ij}}}{K}$, where *K* is the number of command sequences per user. The false alarm rate for the combination *i* is $\frac{1}{N^{\text{int}}}\sum_{j=1}^{N^{\text{int}}}\frac{{\text{FA}}_{\text{ij}}}{K}$, where *N^int^* is the number of internal users. Therefore, the average false alarm rate is:

(9) $$\text{FAR}=\frac{1}{C}\sum_{i=1}^{C}\frac{1}{N^{\text{int}}}\sum_{j=1}^{N^{\text{int}}}\frac{{\text{FA}}_{\text{ij}}}{K}$$

where $C=\left(\begin{array}{c} N^{\text{all}}\\ N^{\text{int}} \end{array}\right)$ is the number of combinations of users, and *N^all^* is the total number of users.

Next, we formulate the average missed alarm rate for internal and external users. Let ${\text{MA}}_{\text{ij}}^{\text{int}}$ be the number of missed alarms for internal users in the combination *i* with the internal user *j*. Then, the missed alarm rate for the combination *i* and the user *j* is $\frac{{\text{MA}}_{\text{ij}}^{\text{int}}}{\left(N^{\text{int}}-1\right)K}$, where (*N^int^* - 1)*K* is the total number of the other user's command sequences. The missed alarm rate for the combination *i* is $\frac{1}{N^{\text{int}}}\sum_{j=1}^{N^{\text{int}}}\frac{{\text{MA}}_{\text{ij}}^{\text{int}}}{\left(N^{\text{int}}-1\right)K}$. Therefore, the average missed alarm rate for internal users is:

(10) $${\text{MAR}}^{\text{int}}=\frac{1}{C}\sum_{i=1}^{C}\frac{1}{N^{\text{int}}}\sum_{j=1}^{N^{\text{int}}}\frac{{\text{MA}}_{\text{ij}}^{\text{int}}}{\left(N^{\text{int}}-1\right)K}$$

Let ${\text{MA}}_{\text{ij}}^{\text{ext}}$ be the number of missed alarms for external users in the combination *i* with the internal user *j*. As described above, the average missed alarm rate for external users is:

(11) $${\text{MAR}}^{\text{ext}}=\frac{1}{C}\sum_{i=1}^{C}\frac{1}{N^{\text{int}}}\sum_{j=1}^{N^{\text{int}}}\frac{{\text{MA}}_{\text{ij}}^{\text{ext}}}{\left(N^{\text{all}}-N^{\text{int}}\right)K}$$

where *N^all^* – *N^int^* is the number of external users.

In general, there is a tradeoff between the false alarm rate and the missed alarm rate. The relation of these rates can be described visually by a receiver operating characteristic (ROC) curve [23], which is a parametric curve generated by varying the threshold *Th* from 0% to 100%, and computing these rates at each threshold *Th*. The ROC curve of internal user detection is described by a trajectory for (*FAR, MAR*^int^), and the ROC curve of external user detection is described by a trajectory for (*FAR, MAR^ext^*). Both curves start from the upper left and go toward the lower right, varying threshold *Th* from 0% to 100%. The lower and further left the curve, the better. The ROC curve of random discrimination is almost equal to a diagonal line from the top left corner to the bottom right corner. In addition, the area under the ROC curve is a scalar measure for ROC analysis. The area under the ROC curve enables quantitative comparison of multiple ROC curves [23].

## 5. Experiments and Discussion

### 5.1. Experimental Data and Configuration

We used Schonlau's masquerader data (http://www.schonlau.net/), which consists of 50 files, one for each user. Each file contains 150 command sequences generated with a process accounting acct. Each command sequence consists of 100 commands. Based on Schonlau's works [7], the first 50 command sequences for each user are the training data for constructing a profile. The next 100 command sequences are test data for evaluating the performance of masquerader detection. Due to the difficulty of collecting external user data, the user data set is logically divided into internal and external user data. Only internal user data are used to construct profiles as training data, while both internal and external user data are used to evaluate their detection accuracy as test data. The masquerader behavior is simulated by testing one user's command sequence against another user's profile. Furthermore, we should evaluate all combinations of internal users among the 50 users in Schonlau's data because the performance of masquerader detection would depend on the combinations. However, as the total number of combinations is very high, for each experiment, we used 1,000 combinations chosen randomly from among all possible combinations. Hence, each point on the ROC curve is an average over 1,000 combinations.

### 5.2. Analysis of Detection Accuracy

#### 5.2.1. Comparison of Detection Methods

**Figure 3.**
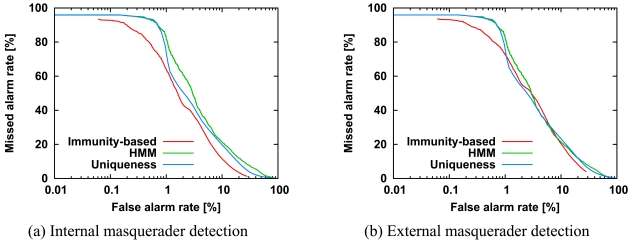
ROC curves for detection of internal masqueraders (a) and external masqueraders (b) using our immunity-based method, the single-sensor HMM method and the uniqueness method. The parameters are as follows: *L* = 100, *K* = 100, *N* = 10, *δ* = *e*^−200^, *N^int^* = 20, *N^ext^* = 25, *C* = 1,000. The number of internal masqueraders is 25, and the number of external masqueraders is the same as that of internal masqueraders. The horizontal axis is a logarithmic scale, so we can focus on the missed alarm rate at the low false alarm rate.

We investigated the detection accuracy of our immunity-based method in comparison with two single-sensor methods, *i.e.*, the HMM method [2,3] and the uniqueness method [4]. The single-sensor HMM and uniqueness methods are among the best methods described previously [7,16]. Figure 3 shows the ROC curves of internal and external masquerader detection for our method, the HMM method, and the uniqueness method. Table 1 shows the statistics of the area under the ROC curves for 1,000 combinations of internal and external users. In each ROC curve, 25 internal users were chosen randomly from among the total of 50 users, and all the others were the external users. All agents were generated from command sequences of internal users.

In Figure 3(a), the ROC curve for our method lies below the other two curves; this indicates that our method outperforms the other two methods. Figure 3(b) indicates that our method outperformed the other two methods with a false alarm rate of less than 1%. These performance improvements were because our method uses not only an activated agent but also the other internal user's agents, while the other two methods use only an activated agent. That is, the use of multiple agents leads to improvement in masquerader detection accuracy. The HMM curve in Figure 3(a) is almost the same as that in Figure 3(b) because the single-sensor method of the HMM approach is independent of the combination of internal and external users. The uniqueness method is slightly better at detecting internal masqueraders than external masqueraders, because the uniqueness method is based on the relative difference of command frequency for internal users.

**Figure 4.**
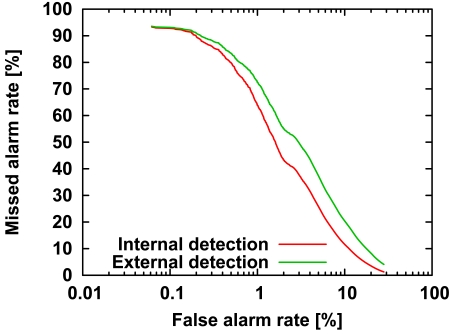
Comparison of ROC curves for detection of internal masqueraders and external masqueraders. The curves are the same as those in Figure 3. The horizontal axis is a logarithmic scale, so we can focus on the missed alarm rate at the low false alarm rate.

Comparing the internal and external masquerader detection rates of our method, as expected, the immunity-based method was better at detecting internal than external masqueraders (Figure 4). As mentioned in Section 4, the differences in the ROC curves would be due to the lack of information on external masqueraders.

#### 5.2.2. Analysis of the number of internal users

**Figure 5.**
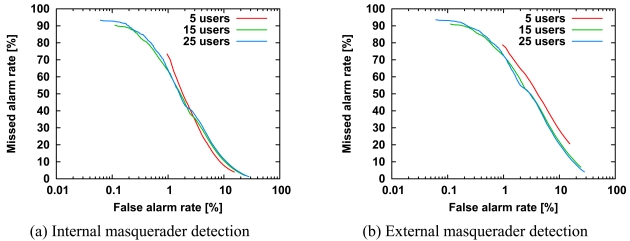
ROC curves for the detection of internal masqueraders (a) and external masqueraders (b) for *N^int^* = 5, 15 and 25 internal users. All other parameters are the same as those in Figure 3. The horizontal axis is a logarithmic scale, so we can focus on the missed alarm rate at the low false alarm rate.

We investigated the effects of the number of internal users on detection accuracy. Figure 5 shows ROC curves for 5, 15, and 25 internal users, respectively, while keeping the number of external users at 25. The difference in length of the ROC curves is related to the number of agents, because discrimination between normal and abnormal is based on *Th* and the scores of all agents as described in Section 3.3.

In Figure 5, it might appear that the ROC curves largely overlap with each other, but the detection accuracy is slightly proportional to the number of internal users in Figure 5(b). That is, an increase in number of internal users may improve the detection accuracy of external masqueraders, although the detection accuracy is almost independent of the number of internal users. In Figure 5(b), the improvement of detection accuracy may be related to the diversity of agents.

#### 5.2.3. Analysis of the number of agents

**Figure 6.**
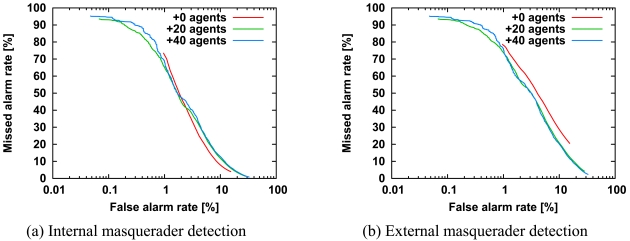
ROC curves for detection of internal masqueraders (a) and external masqueraders (b) for the number of additional agents, keeping *N^int^* = 5 and *N^ext^* = 5. All other parameters are the same as in Figure 3. The horizontal axis is a logarithmic scale, so we can focus on the missed alarm rate at the low false alarm rate.

We conceived the addition of agents to diversify agents inspired by the diversity of the immune system. To add new agents, we virtually divided 50 agents into internal agents, additional agents, and other agents randomly. The internal agents correspond to internal users. The additional agents correspond to neither internal users nor external users, *i.e.*, external masqueraders. The other agents correspond to external masqueraders, and they are not used in detection evaluation. Briefly, the additional agents are constructed from actual command sequences of users other than internal users and external masqueraders. In the test evaluation, the additional agents compute the score for the most recently executed command sequence and present their own score to the activated agent as well as the internal agents, but the additional agents are not activated because they do not have their own account. Figure 6 shows ROC curves of internal and external masquerader detection for 0, 20, and 40 additional agents. The number of internal users is five, and the number of external users is five. In Figure 6(a), all curves are similar to each other, whereas in Figure 6(b), the detection accuracy is improved. Briefly, the addition of agents improves the detection accuracy of external masqueraders. In addition, the improvement in Figure 6(b) is similar to that in Figure 5(b). That is, the addition of agents seems to have the same effect as an increase in number of internal users.

We now consider why only external masquerader detection is improved in Figure 6(b). Internal masquerader detection is improved if an agent that is specialized to recognize a specific internal user computes the highest score of all the agents against command sequences of the specific internal user corresponding to the agent. In contrast, external masquerader detection is improved if any additional agent computes the highest score of all the agents against command sequences of external masqueraders. Note that the additional agents must not compute the highest score against any command sequences of internal users. Therefore, the addition of agents improves only external masquerader detection, while internal masquerader detection remains almost unchanged.

Masquerader detection would be best achieved by adding agents specialized to recognize an external masquerader. Suppose that we add these agents in advance so that there is a one-to-one correspondence between agents and masqueraders. The detection accuracy of external masqueraders would be similar to that of internal masqueraders. Briefly, addition of profiles should improve the detection accuracy of external masqueraders to the extent of that of internal masqueraders. In all the experiments, ROC curves of external masqueraders deviated from those of internal masqueraders, suggesting that the addition of agents could improve the detection accuracy of external masqueraders.

#### 5.2.4. Discussion of artificial diversification

In the experiments shown in Figure 6, we added agents whose profiles were constructed from actual command sequences. In actual use, however, it would be necessary to generate additional agents, *i.e.*, external user-specific agent, without actual masqueraders' command sequences, as it is difficult to gather actual command sequences from masqueraders. One simple method of generating the external user-specific agents is random generation. However, random generation did not improve the detection accuracy because the scores computed from randomly generated profiles were remarkably low. Hence, the profile should be somewhat similar to internal user profiles to increase the score, but the score must not exceed that of the activated agent against command sequences of the user corresponding to the activated agent.

This is similar to the mechanism involved in training of T-cells in the thymus, *i.e.*, positive selection and negative selection. In the thymus, T-cells generate diverse T-cell receptor repertoires by cell division with gene rearrangement. The newly generated T-cells that recognize self major histocompatibility complex (MHC) molecules are *positively* selected. They have various affinities for binding self MHC molecules. However, many of these T-cells are potentially harmful because their receptors have high affinity for a complex of self peptide and a self MHC molecule. These T-cells are *negatively* selected and eliminated. The surviving T-cells are exported from the thymus. They have weak affinity for self MHC molecules, which means they have the potential not only to recognize self MHC molecules, but also to recognize a foreign peptide complex.

This similarity prompted us to apply a similar mechanism to the generation of external user-specific agents. We used a diversification mechanism to generate external user-specific agents that compute a low score for legitimate users' command sequences, and a high score for external users' command sequences. The flow of the mechanism is as follows:

Step 1. A *base HMM* is constructed by averaging the HMMs of internal users. Note that the statistical properties of the *base HMM* are similar to those of an HMM estimated from all the command sequences of internal users.
Step 2. Artificial command sequences are generated using the *base HMM*. The command sequences can consist of the characteristics of the average user.
Step 3. An HMM is estimated from the artificial command sequences, and an agent associated with the HMM is generated.
Step 4. If the score computed by the agent is lower than the minimum of the scores computed by all internal user-specific agents, the agent is removed, *i.e.*, all the other agents are *positively* selected (positive selection), and the process for generating the agents returns to Step 2. If the score computed by the agent is higher than the maximum of the scores computed by all internal user-specific agents, the agent is removed, *i.e.*, all the others are *negatively* selected (negative selection), and the process returns to Step 2.
Step 5. The agent is incorporated into the detection system and the process returns to Step 2 until the number of incorporated agents exceeds a specified number (e.g., 10 in our experiment). The incorporated agent could have the potential to recognize command sequences of external masqueraders.

**Figure 7.**
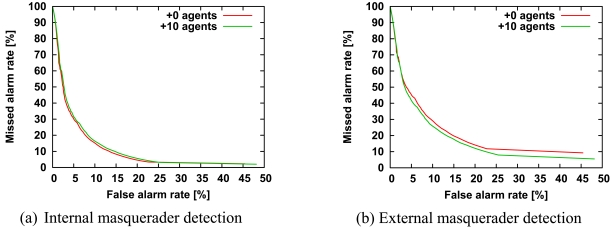
ROC curves of internal masquerader detection (a) and external masquerader detection (b) for 10 external user-specific agents and no additional agents. The parameters are as follows: *N^int^* = 10, *N^ext^* = 10. All other parameters are the same as those in Figure 3. The length of the command sequences generated by the base HMM is the same as that in the training data. The number of command sequences is 25.

According to these steps, we generated more than 10 external user-specific agents and evaluated how the detection accuracy was improved. The number of internal users is 10, and the number of external users is 10. The base HMM is averaged over the HMMs of all internal users. The length of the command sequences generated by the base HMM is the same as that used in the training data. The number of the command sequences is 25. Figure 7 shows the ROC curves of 10 external user-specific agents and no additional agents. The detection accuracy for more than 10 external user-specific agents is almost the same as that for 10 external user-specific agents.

The curve with 10 external user-specific agents was better at detecting external masqueraders, but not quite as good at detecting internal masqueraders. Therefore, the diversification mechanism is good at tightening the security for external masqueraders rather than internal masqueraders.

The detection accuracy of internal masquerader detection deteriorates when at least one external user-specific agent computes a score higher than that of the activated agent against command sequences of internal users. One reason for the external user-specific agent computing a higher score is that the upper threshold for negative selection is high. To avoid this, the threshold could be lowered, but this would be at the expense of accuracy for detecting external masqueraders. Another reason is that the activated agent computes a very low score when the user uses commands not included in the training data.

With regard to Figure 7(b), the reason why the detection accuracy of external masqueraders is improved is that any external user-specific agent computes the highest score of all the agents against command sequences of external masqueraders. Briefly, the external user-specific agents can recognize unknown external users.

#### 5.2.5. Analysis of HMM parameters

The HMM would have some parameters that affect the detection accuracy. The variable parameters of the HMM are the number *N* of hidden states, the minimum probability δ of the command probability distribution, the length *L* of test sequences, and the number *K* of training command sequences.

**Figure 8.**
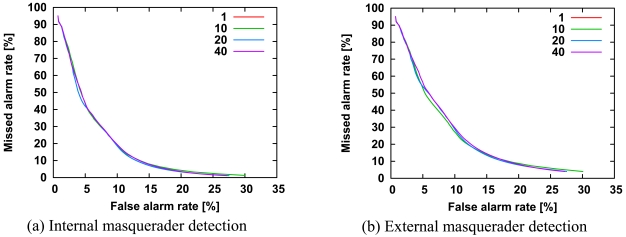
ROC curves for internal masquerader detection (a) and external masquerader detection (b) for the number of hidden states. All other parameters are the same as those in Figure 3.

We investigated the number of hidden states from 1 to 50. All the other parameters are the same as those in Figure 3. As shown in Figure 8, all curves largely overlapped with each other. In contrast to our expectations, the detection accuracy is independent of the number of hidden states. Our expectations were based on our previous report that detection accuracy for the single-sensor HMM method was positively correlated with the number of hidden states [3]. This may have been due to differences in experimental data between our previous study and the present study. The experimental data provided by Schonlau depends the frequency of specific commands rather than that of chains of specific commands in contrast to the experimental data used in our previous study. We can set the number of hidden states to 1, which has the lowest computational cost, because the detection accuracy is independent of the number of hidden states.

**Figure 9.**
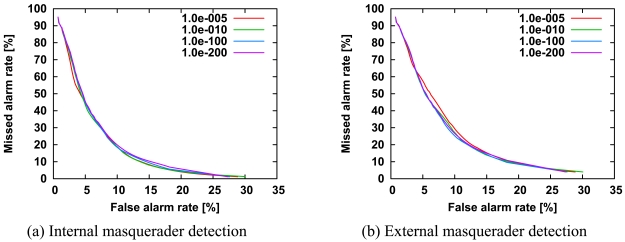
ROC curves of internal masquerader detection (a) and external masquerader detection (b) for the minimum probability. All other parameters are the same as those in Figure 3.

The minimum probability allows us to control the score of test sequences that include commands not seen in the training data. In fact, the significant small probability could improve the detection accuracy of the single-sensor method of the HMM [3]. However, similar to the number of hidden states, the minimum probability is unrelated to detection accuracy (Figure 9). This may be because of differences in discrimination methods; in the immunity-based method, discrimination is based on the relative evaluation of scores computed by multiple sensor agents, while in the single-sensor method discrimination is based only on the one score. Briefly, the relative evaluation of scores negates the effect of the minimum probability.

**Figure 10.**
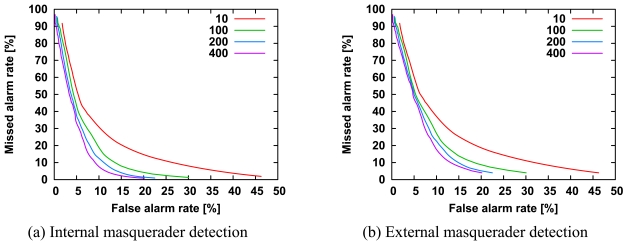
ROC curves for detection of internal masqueraders (a) and external masqueraders (b) according to the length of test sequences. All other parameters are the same as those in Figure 3.

Similar to the length of command sequences, the ROC curves did not overlap with each other (Figure 10). As expected, the longer the length, the greater the improvement in detection accuracy.

**Figure 11.**
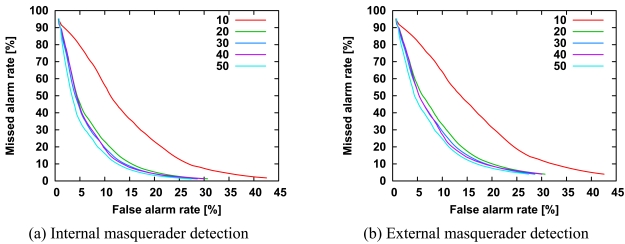
ROC curves for detection of internal masqueraders (a) and external masqueraders (b) for the number of training sequences. All other parameters are the same as those in Figure 3.

Figure 11 shows the ROC curves of internal masquerader detection and external masquerader detection according to the number of training command sequences. Similar to the length of command sequences, the greater the number of command sequences, the greater the improvement in detection accuracy. It is noteworthy that detection accuracy is improved markedly by varying the number of training sequences from 10 to 20.

## 6. Conclusions

A new immunity-based anomaly detection system with sensor agents for detecting masqueraders was developed and evaluated. This immunity-based system utilizes multiple sensors, which can improve detection accuracy compared to single-sensor methods. In addition, an evaluation framework for the immunity-based anomaly detection system was proposed. The evaluation framework is capable of evaluating the differences in detection accuracy between internal and external masqueraders.

Evaluation results can be summarized as follows:

- The new immunity-based method outperformed the single-sensor HMM and uniqueness methods.
- An increase in number of internal users slightly improved the detection accuracy of external masqueraders, although the detection accuracy was almost independent of the number of internal users.
- The addition of agents improved the detection accuracy of external masqueraders, although the detection accuracy was almost independent of the number of additional agents.
- The proposed diversification mechanism is useful for tightening security against external masqueraders rather than internal masqueraders.

We are currently developing an adaptation mechanism that will make it possible to update a profile, *i.e.*, to reestimate the parameters of the HMM for newly executed command sequences that the immunity-based system classified as “self”. Updating of a profile would enable agents to adapt to a change in user behavior, and would be expected to improve detection accuracy.

## Figures and Tables

**Table 1.** Statistics on the area under the ROC curves for detection of internal masqueraders and external masqueraders using our immunity-based method, the single-sensor HMM method and the uniqueness method.

Method	Masquerader	Mean	Standard deviation	95% confidence interval
Immunity-based	Internal	0.04456	0.01001	0.04394 – 0.04518
HMM	Internal	0.09442	0.01537	0.09347 – 0.09538
Uniqueness	Internal	0.06435	0.01002	0.06373 – 0.06435

Immunity-based	External	0.07375	0.01009	0.07312 – 0.07438
HMM	External	0.09481	0.01526	0.09386 – 0.09575
Uniqueness	External	0.07839	0.01004	0.07776 – 0.07902
